# What do we currently know about Novichoks? The state of the art

**DOI:** 10.1007/s00204-022-03437-5

**Published:** 2022-12-30

**Authors:** Maciej Noga, Kamil Jurowski

**Affiliations:** 1 Laboratory of Innovative Toxicological Research and Analyzes, Institute of Medical Studies, Medical College, Rzeszów University, Al. Mjr. W. Kopisto 2a, 35-959 Rzeszów, Poland; 2 Department of Regulatory and Forensic Toxicology, Institute of Medical Expertises, Ul. Aleksandrowska 67/93, 91-205 Łódź, Poland

**Keywords:** Novichoks, Toxicology, Organophosphate, Nerve agents, Chemical warfare agent

## Abstract

Novichok is the name given to the group of nerve agents created stealthily in the later phases of the Cold War by the Soviet Union. Constitute the fourth generation of chemical warfare agents; like other nerve agents, they are organophosphorus compounds designed to be incurable and undetectable. The mechanism of action is based on the non-competitive and irreversible inhibition of acetylcholinesterase. Due to their enormous toxicity, Novichoks have become attractive targets for terrorists. However, little information is known about the identity of nerve agents. Furthermore, these compounds have never been submitted to the Chemical Weapons Convention. Our article aspires to provide a general overview of Novichoks knowledge. As part of this, we reviewed the available literature data to answer the question, what are Novichoks? In addition to the physical and chemical properties of A-agents, synthesis, mechanism of action, and toxicity of nerve agents were also reviewed. We hope that this review will highlight the tremendous threat posed by nerve agents and will inspire further studies on the interdisciplinary aspects of these compounds.

## Highlights

- Novichoks, an extremely life-threatening nerve agents.
- General overview of the information on Novichoks: physical and chemical properties, mechanism of action and toxicity data.
- Novichoks should be treated as a separate group of chemical warfare compounds due to their hazardous properties.

## Introduction

**Fig. 1:**
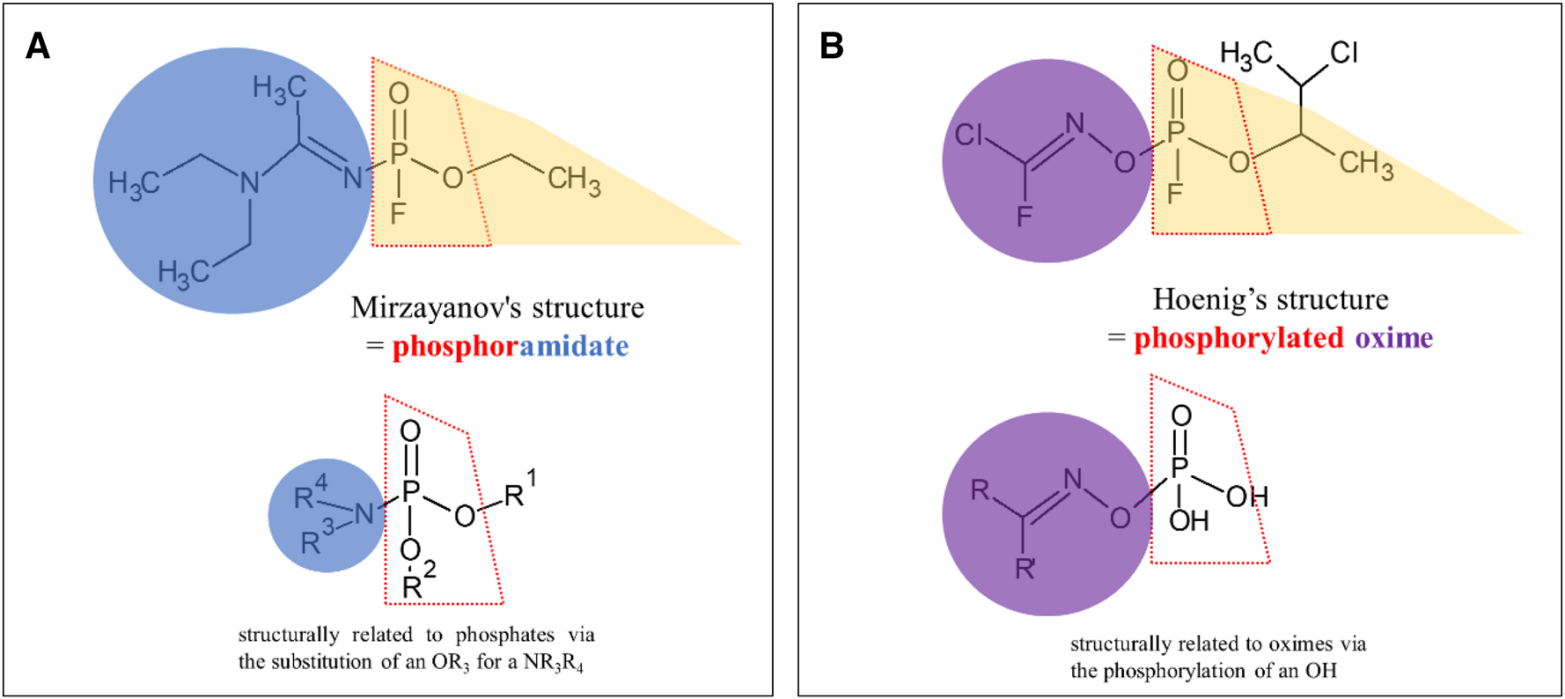
Proposed chemical structures of the Novichok agent as A-234: **A** Mirzayanov’s structure as a phosphoramidate, and **B** Hoenig's structure as a phosphorylated oxime

A Novichok agent (Russian: Hoвичóк, which means ‘newcomer’ in Russian) (Hussain and Sharma 2019) can be defined as a hypothetical group of nerve agents; some of these can be classified as binary (two inert substances combined prior to delivery to create the active nerve agent) chemical weapons. It is assumed that the Novichok agents come from the testimony and memoirs of Vil S. Mirzayanov, the Director of the Department of Counteraction against Foreign Technical Intelligence at the Russian State Union Scientific Research Institute for Organic Chemistry and Technology (GosNIIOKhT) (Mirzayanov 2008). Mirzayanov disclosed information about initiating a secret Soviet chemical weapons initiative to develop Novichok agents. The first three of these, Substance-33, A-230 and A-232, were probably produced in a GosNIIOKhT facility in Russia using an organophosphate structural backbone [R_1_–P(=O)(R_2_)–OR_3_; R_1_ = amide/oxime; R_2_ = fluorine; R_3_ = alkyl, alkoxy, aklylamino] (Smithson et al. 1995). From a chemical point of view, Novichok compounds are postulated to be organophosphates containing a dihaloformaldoxime moiety (Gupta 2015). Mirzayanov proposed the first chemical structure proposition for Novichoks (including A-234 as a phosphoramidat)—Fig. 1A.

However, (at the same time) Hoenig (2007) and Ellison (2007) proposed alternative structures for Novichoks as phosphorylated oxime—Fig. 1B. Therefore, data on these hazardous materials are still buried in mystery (Nepovimova and Kuca 2020; Franca et al. 2019). The only available data come from the interviews and articles of Mirzayanov et al. (Mirzayanov 2008; Karev 2009), but in the opinion of most scientists, it is not an entirely reliable data source (it seems that Hoenig’s and Ellison’s version is the most realistic). Currently, studies about Novichoks are rare and have only recently started to emerge (Imrit et al. 2020). However, Chai et al. (2018) and Harvey et al. (2020) noted that there is some consensus on the phosphoramidate nature of A-series nerve agents. So, the question is ‘What exactly are these dangerous substances?’ Perhaps, because of the high reactivity of these substances, predictions would be appropriate using in silico toxicology tools like QSAR? Only a few studies are available in the scientific literature on this topic.

An interesting fact is that Novichoks were designed to be undetectable by standard North Atlantic Treaty Organization (NATO) chemical detection equipment (Nepovimova and Kuca 2018), so their identification is much more difficult. For this purpose, a beneficial methodology is based on ultrasensitive detection of the Novichok nerve agent A-232 using vibrational spectroscopy (Tan et al. 2019).

There is no doubt that the chemical attack in Salisbury, Wiltshire, United Kingdom, on 4 March 2018 exposed a case of acute poisoning (Sergei Skripal and his daughter, Yulia) by this type of compound (probably a Novichok nerve agent, A-234) (Bhakhoa et al. 2019; Haslam et al. 2022). However, it was not only an exceptional case, because on 30 June 2018 in Amesbury, Wiltshire, United Kingdom, the poisoning of two British nationals occurred by a Novichok nerve agent of the same kind used in Salisbury (13 km away) (Haslam et al. 2022). Two years later (on 20 August 2020), a previously healthy 44-year-old man suddenly became confused and began sweating heavily on a domestic flight to Russia approximately 10 min after departure (Steindl et al. 2021). Two weeks later, the German government announced that a laboratory of German armed forces designated by the Organization for the Prohibition of Chemical Weapons (OPCW) had identified an organophosphorus nerve agent from the Novichok group in blood samples from this patient (Steindl et al. 2021). The examples confirmed the effects of acute poisoning by Novichok agents, indicating the probability of the presence of these substances.

As noted above, in the scientific literature, only a few articles are dedicated to the specific properties of the chosen Novichok agents; however, there is still a lack of a comprehensive review of what we know about Novichoks. To our knowledge, this is the first comprehensive and actual review of Novichoks that includes the current state of knowledge from an interdisciplinary perspective.

## Materials and methods

### Search for publications on Novichok data

For the critical review of Novichoks data: Scopus, Google Scholar, and Web of Science were applied as the primary repositories for finding published references on this topic. It should be noted that the data collection process involved searching mentioned scientific sources but also 'grey' literature (V. S. Mirzayanov, State Secrets: An Insider’s Chronicle of the Russian Chemical Program, Outskirts Press, Inc., 2008; general commercial, trade body, and industrial collections), using theoretical and empirical data. Different combinations of the following principal terms were used: Novichok; nerve agents; A-series compounds; Mirzayanoy; Sergei Skripal; Alexei Navalny; organophosphate; A-234; A-230; A-232; *N*-[ethoxy(-fluoro)phosphoryl]-*N*,*N*-diethylethanimidamide; VX; CBRN. All available sources (*n* = 52 articles and related content) were analysed. Only articles/works related to Novichok agents were considered to filter the sources retrieved.

### Classification and presentation of the results

A different Novichoks agent has been used as the target specimen, up to 14 species in the reviewed works. For better readability, data and characteristics of the described Novichoks were presented in appropriate sections of this work, i.e. physical and chemical properties, synthesis, mechanism of action, and toxicity.

## Physical and chemical properties of Novichoks

There is no doubt that Novichok agents are extremely hazardous xenobiotics; therefore, the first step should be the physical and chemical characterisation of these substances. However, very little information is available (Karev 2009; Halámek and Kobliha 2011; Pitschmann 2014). In scientific articles (mainly described by Nepovimova and Kuca 2018, 2020), only a few properties according to three Novichok agents, that is, A-230, A-232, and A-234 (possible A-series nerve agents reported by Hoenig) and other possible Novichoks reported by Ellison (2007) and Patočka (2018) are published.

The first three A-agents reported by Hoenig (A230, A-232, and A-234) are volatile liquids with similar densities (1.414–1.612 g·mL^−1^) and similar boiling points. At low temperatures, A-232 and A-234 do not solidify. A-242 is solid at room temperature, according to Mirzayanov. However, these data are uncertain due to the lack of confirmation of this information in the literature. A-230 is resistant to moisture; however, A-232 is less stable to moisture than A-230. The low vapour pressures stated for A-230, A-232, A-234, and A-242 suggest that they persist indifferently in the environment. Other Novichoks reported by Ellison and Patocka can be considered to be a heterogeneous group because of differences in properties. A significant disadvantage of Novichoks is the lack of balance between volatility and persistence (Nepovimova and Kuca 2018). Table 1 represents available data on the physical and chemical properties of Novichoks reported by Hoenig (A-230, A232 and A-234) and other Novichoks reported by Ellison and Patocka. The volatility parameter for Novichoks (other than A-230, A-232 and A-234) was reported by Ellison and Patock using ‘ppm’ units (Ellison 2007; Patočka 2018). By comparing the volatility of compounds with a known parameter, such as G-agent and V-agent (GB = 2800 ppm, volatile; GD = 520 ppm, volatile; VX = 1.2 ppm, not volatile) with Novichoks listed in Table 1, we determined their volatility.

**Table 1:** Data available on the physical and chemical properties of chosen; based on (Ellison 2007; Mirzayanov 2008; Patočka 2018; Nepovimova and Kuca 2018, 2020)

Novichok	State	Density, g·mL^−1^	Volatility	Melting point, °C	Boiling point, °C	Vapour pressure, Pa	Solubility at 25 °C, g·L^−1^	Log P	Behaviour at low temperature	Stability under moisture conditions
A-230	Liquid	1.612	Volatile	5.56	61–62 [5,10] 298 [X]	2.13	4.823	2.14	Solidifies at low temperature	Resistant to moisture
A-232	n.a	1.515	More volatile than A-230	5.65	70–71 [5,10] 259.92 [X]	1.48	1.775	2.55	Does not solidify at low temperature	less stable against moisture than A-230
A-234	Very viscous liquid	1.414	Poorly volatile	3.06	73–74 [5,10] 264.11 [X]	1.7	0.6512	2.97	n.a	Resistant to moisture
A-242	Solid	n.a	n.a	21.46	284.85 [X]	0.579	1000	0.45	n.a	n.a
	n.a	1.488	Volatile (600 ppm)	−12.33	218.93	18.5	16.74	1.76	n.a	n.a
	n.a	1.510	Volatile (2000 ppm)	−35.05	191.37	73.8	37.64	1.47	n.a	n.a
	n.a	1.424	Highly volatile (10,000 ppm)	−58.28	162.02	304	84.02	1.18	n.a	n.a
	n.a	1.639	Volatile (900 ppm)	48.34	256.81	1.53	9.940	1.34	n.a	n.a
	n.a	1.366	Poorly volatile (200 ppm)	−1.27	237.51	7.04	5.374	2.12	n.a	n.a
	n.a	1.450	Volatile (1000 ppm)	−23.2	211.21	27.4	12.15	1.83	n.a	n.a
	n.a	n.a	Volatile (4000 ppm)	−46.48	183.14	110	27.29	1.54	n.a	n.a
	n.a	1.506	Poorly volatile (200 ppm)	58.85	273.49	0.509	3.157	1.70	n.a	n.a

## Synthesis of Novichoks

Most of the current available information about A-series nerve agents is speculative or comes from uncertain sources that have not yet been confirmed. This state of affairs creates a significant problem for which information may be considered reliable. Due to these divergences, we have included all known A-agent synthesis routes to show the differences between these sources.

**Fig. 2:**
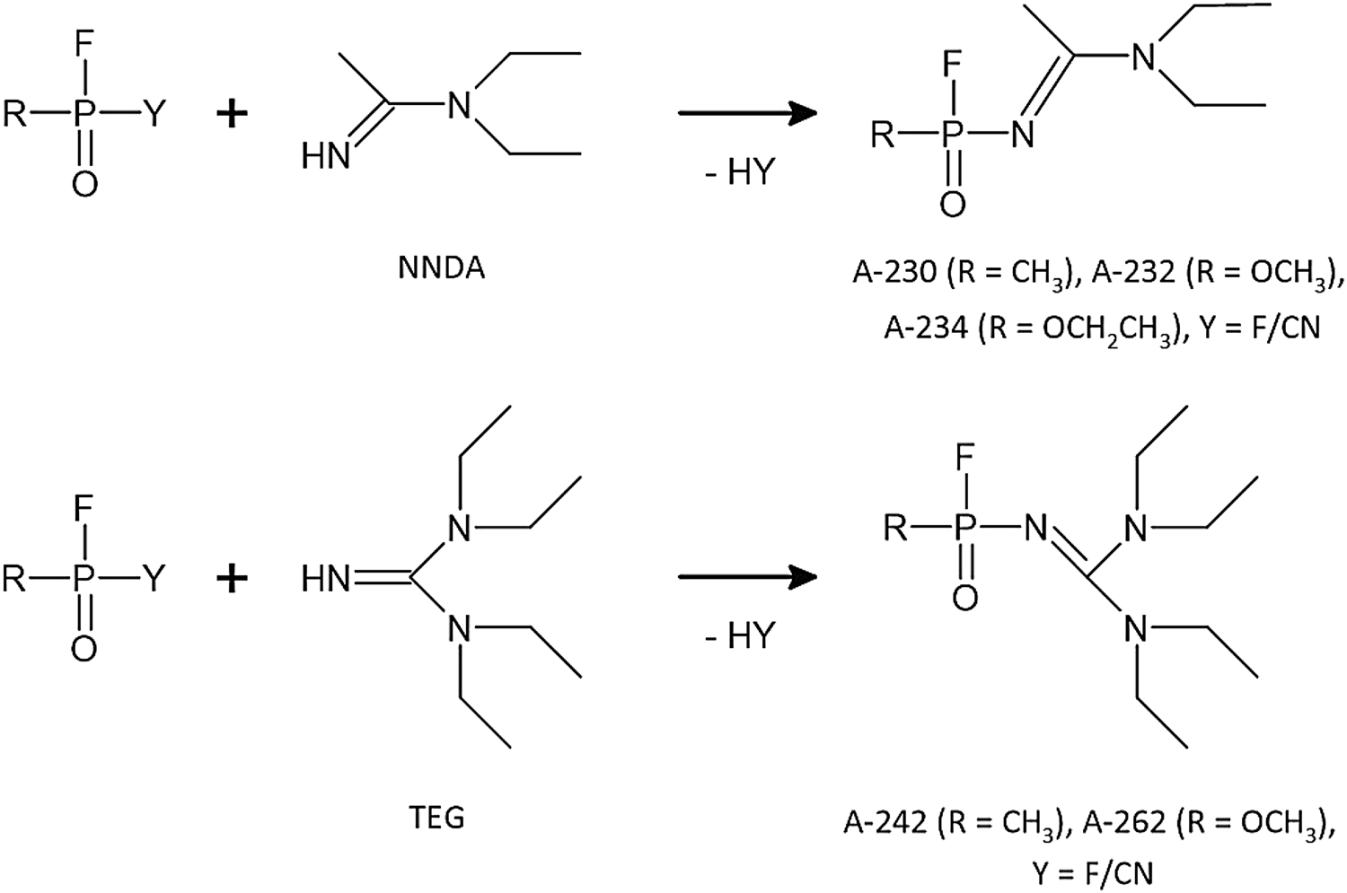
Synthesis routes for the A-series compounds according to Mirzayanov. Group Y is either fluorine (F) or cyanide (CN)

As reported by Mirzayanov, the A-series compounds belong to the phosphoramidate group. Synthesis of compounds with code symbols: A-230, A-232, A-234, A-242, and A-262 (Fig. 2) is described in his book “State Secrets. An Insider's Chronicle of the Russian Chemical Weapons Program” (Mirzayanov 2008). The synthesis route of A-230 is based on the condensation of *N*,*N*-diethylethanimidamide (NNDA) with difluoride (DF). Replacement of DF with *O*-methylphosphonyl difluoride or *O*-methylphosphonylfluorocyanide results in the formation of A-232. A-234 can be obtained using *O*-ethyl phosphonyldifluoride or *O*-ethyl phosphonylfluorocyanide instead of DF. Replacement of NNDA by 1,1,3,3-tetraethylguanidine (TEG) in condensation with DF resulted in the synthesis of A-242. A similar situation occurs when preparing A-262; replacing NNDA with TEG and condensation with *O*-methyl phosphonyldifluoride or *O*-methyl phosphonylfluoro cyanide produces A-262.

**Fig. 3:**
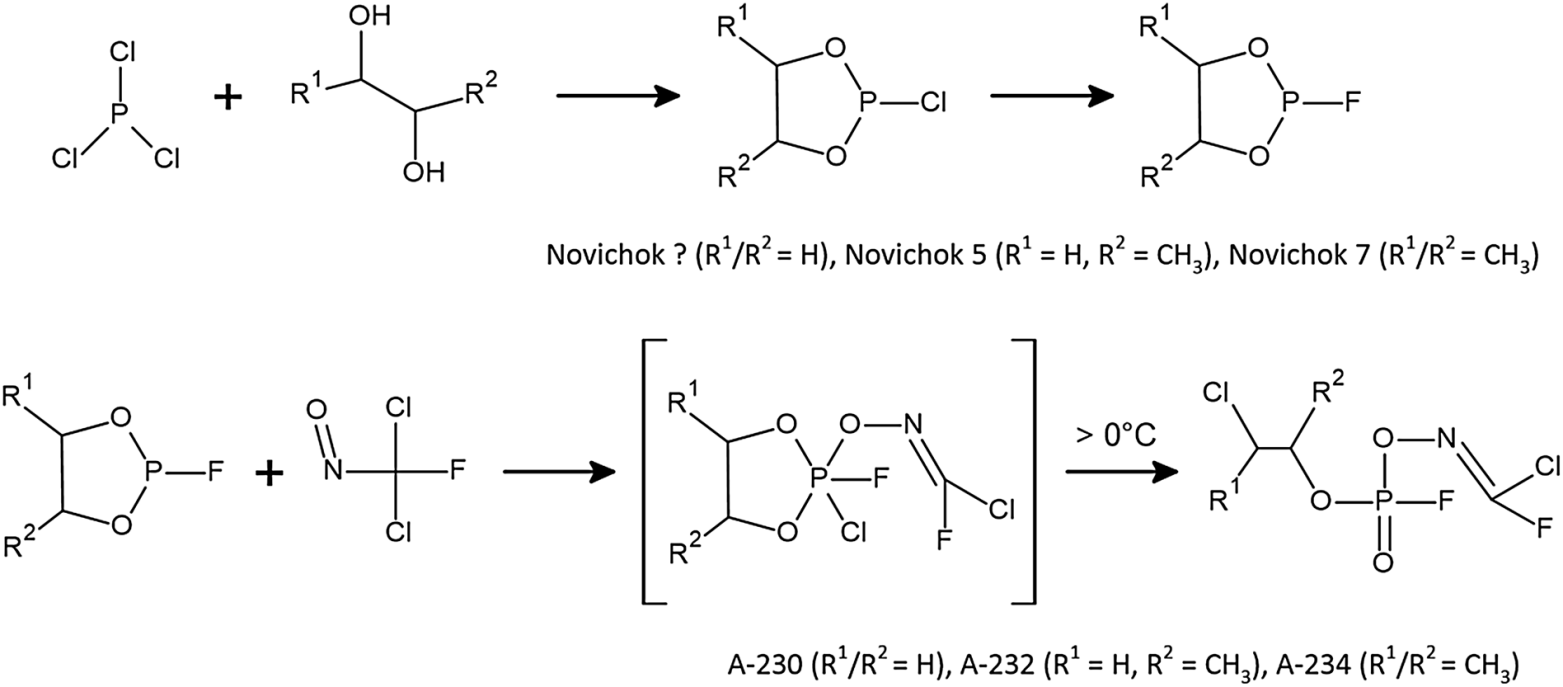
Synthetic pathway leading to phosphorylated oximes, according to Hoenig

A different approach from what Mirzayanov claims is the approach in which A-agents belong to the group of phosphorylated oximes (Halámek and Kobliha 2011; Ellison 2016). In this case, their synthesis will take three steps (Fig. 3). The two initial steps in the synthesis are the preparation of A-agent precursors, referred to as: Novichok?, Novichok 5 and Novichok 7. It consists of the reaction between the phosphorus trichloride with the appropriate diol and the subsequent nucleophilic substitution, in which the chlorine atom is converted into a fluorine atom. The last step in the synthesis of compounds as phosphorylated oximes is the reaction between the 2-fluoro-1,3,2-dioxaphospholanes formed in the previous step and dichloro(fluoro)nitrosomethane. The resulting compound is stable at subzero temperatures (−40 °C). Heating the product facilitates the nucleophilic attack by chloride anion. Results in the opening of the phospholane ring with the transfer of the chlorine atom, creating the appropriate Novichok (Halámek and Kobliha 2011; Hoenig 2007). In addition to phosphorus chlorides or oxychlorides, many other intermediates in phosphorus chemistry used in the pesticide, plasticizer or detergent industry can be used to synthesize A-series compounds as substrates (Nepovimova and Kuca 2018).

**Fig. 4:**
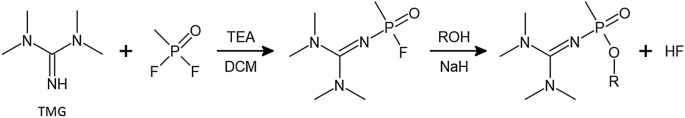
Synthesis of *O*-Alkyl *N*-[bis(dimethylamino)methylidene]-*P*-methylphosphonamidates; based on Hosseini et al. (2016)

A group of Iranian scientists published an article in which they presented a laboratory method for the synthesis of *O*-alkyl *N*-[bis(dimethylamino)methylidene]-*P*-methylphosphonamidates (Fig. 4) (Hosseini et al. 2016). The primary compound described was an analogue of Mirzayanov’s A-242. The analogue had methyl substituents on nitrogen atoms instead of ethyl substituents. The compound was formed by mixing a solution of DF in dichloromethane (DCM) with a solution of 1,1,3,3-tetramethylguanidine (TMG) in triethylamine (TEA) and DCM. They also characterised several derivatives of this compound in which *O*-alkyl (methyl, ethyl, isopropyl) and *O*-aryl (phenyl and 2,5-dimethylphenyl) groups were substituted with the fluorine atom. They were obtained by dropping a solution of the compound previously described in DCM into a previously prepared solution of the appropriate alcohol (ROH) and sodium hydride (NaH) in the same solvent (Hosseini et al. 2016).

## Mechanism of action of Novichoks

**Fig. 5:**
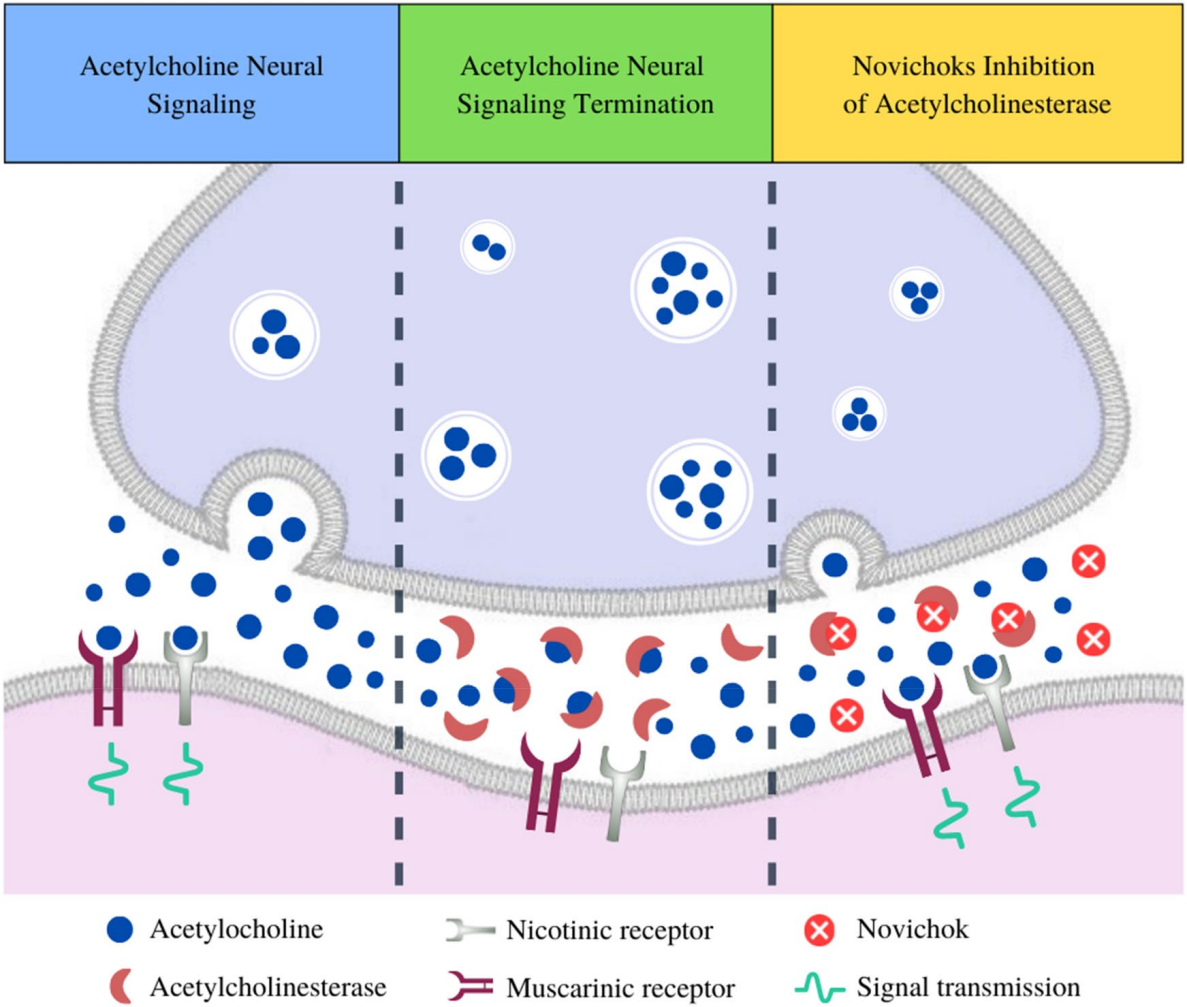
The acetylcholine pathway in cholinergic synapses and interaction with the Novichok nerve agent. ACh neural signalling begins while presynaptic neurons release ACh vesicles that will bind to receptors (muscarinic and nicotinic) in postsynaptic neurons. The cascade ends when AChE cleaves free ACh into acetate and choline. Novichok competitively binds to AChE and inhibits ACh cleavage, thus maintaining constant signal transmission

A-agents can bind to acetylcholinesterase (AChE) and inhibit acetylcholine (ACh) metabolism (Chai et al. 2018). AChE catalyses the hydrolysis of the ACh neurotransmitter to acetate and choline (Hoenig 2007). Hydrolysis of carboxylic esters occurs at the active site of AChE, specifically in the Ser–His–Glu triad (Chai et al. 2018; Nepovimova and Kuca 2018). Under physiological conditions, the hydrolysis of ACh is rapid, which reduces its concentration in neuronal cholinergic synapses and neuromuscular junctions (Dvir et al. 2010). The mechanism of the action of Novichok on the nervous system is through the active site of AChE (Ser–His–Glu triad) (Hoenig 2007). Based on the rapid attack of the hydroxyl groups in serine, which act as a nucleophile on the phosphate groups of the compound (Mercey et al. 2012). Thus, fluoride ions were released that form a phosphorylated enzyme complex. The effect of creating a covalent bond between phosphorus atoms and AChE serine is to slow the hydrolysis of ACh (from hours to days) (Korabecny et al. 2014) (Fig. 5).

There is a circumstance, such as ageing of the AChE enzyme, which causes its inactivation. Any therapy cannot restore it to an active state (Sharma et al. 2015). AChE inhibited by A agent is ageing rapidly. The ageing half-time of A-230 and soman is relatively similar (2–4 min) (Sirin et al. 2012). The aged form of the enzyme was created by rapid hydrolysis of the =N–O– bond in the Novichok-AChE adduct. The phosphonic oxyanion creates a salt bridge with protonated histidine, stabilising the conjugate (Nepovimova and Kuca 2018). Due to rapid ageing of AChE and the weak partial positive charge, reactivation of AChE inhibited by A-agents is a relatively thorny task. Therefore, symptomatic treatment or the dispensing of bioscavengers is an effective therapy (Nepovimova and Kuca 2020). The molecule dispensed in this way would bind to the A agent and thus it would not be able to reach the AChE tissue and cause symptoms of intoxication (Bajgar et al. 2009). An example of such a molecule is butyrylcholinesterases (BChE; E. C. 3.1.1.8). BChE can detoxify all types of A-agents, making it a universal treatment approach (Bajgar et al. 2009).

## Toxicity of Novichoks

**Fig. 6:**
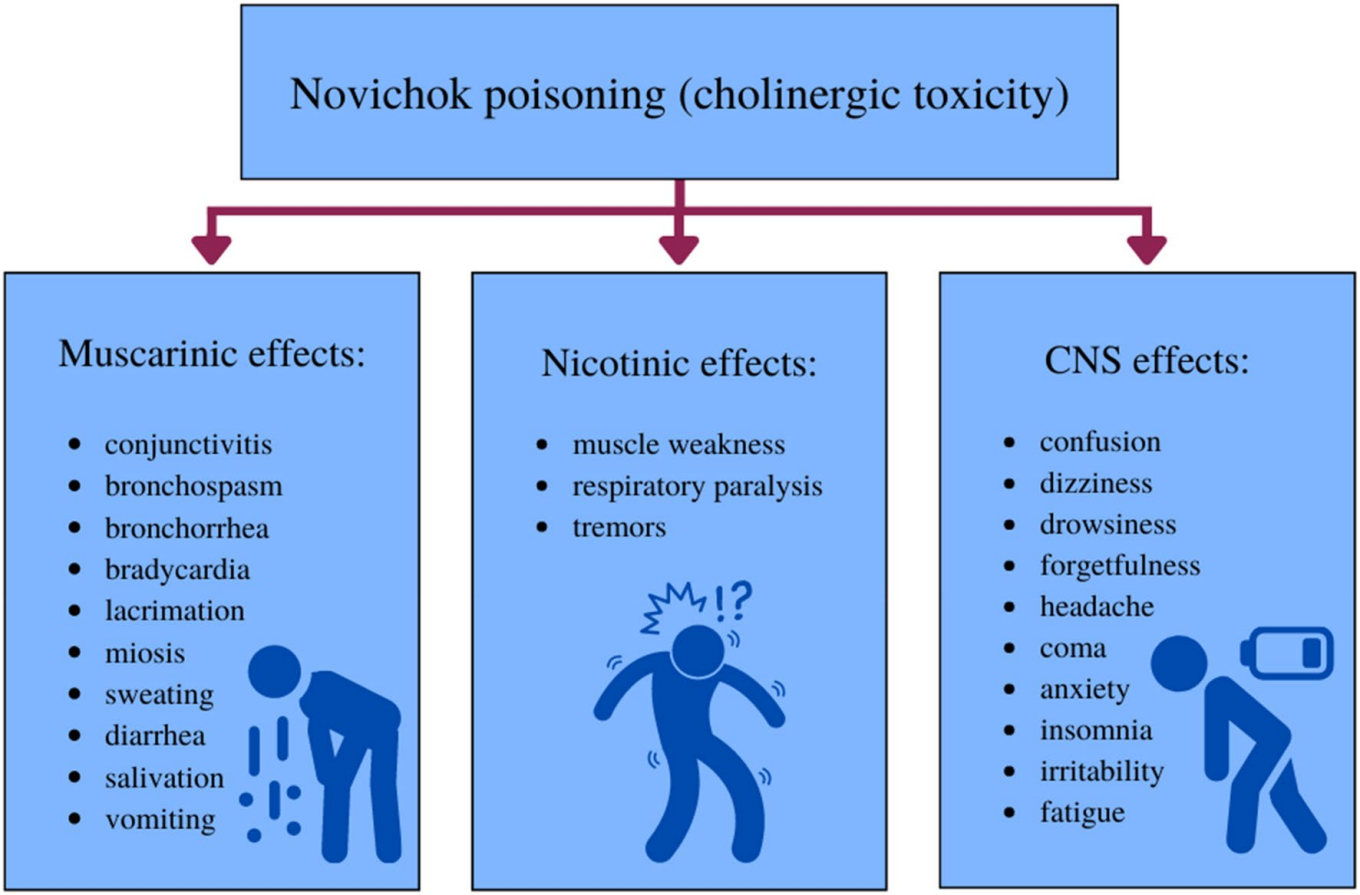
Muscarinic, nicotinic, and central nervous system (CNS) effects of acute Novichok poisoning

Information on the toxicity of A-series nerve agents is minimal. Regarding the exposure routes, depending on the structure of the A-agents: liquid or solid, they can be absorbed through the skin or inhaled (Korabecny et al. 2014). Exposure to A-series nerve agents depends directly on the dose absorbed into the body. These are three types of toxic reactions due to disturbance of AChE activity: muscarinic, nicotinic and central nervous system (Chai et al. 2018; Kloske and Witkiewicz 2019; Kloske 2020) (Fig. 6).

Furthermore, A-agents may be associated with peripheral sensory nerves, and in high doses and with sustained contact, they cause peripheral neuropathy (Gupta 2015). During the hydrolysis of ACh, AChE is also involved in haematopoiesis and the development of nerves and muscles (Colovic et al. 2013). The effect of A-series nerve agents other than those mentioned above is the induction of irreversible neuropathy, so treatment with traditional antidotes for paralytic convulsive agents may be ineffective (Kloske 2020).

According to Mirzayanov, A-230 is 5–8 times more toxic than VX, while A-232 is ten times more toxic than Soman. Moreover, A-242 and A-262 exceed even A-230 and A-232 in their toxicity, making them the most toxic of the A-series compounds (Mirzayanov 2008). However, there is a lack of adequate studies about this important topic. Carlsen presented completely different data on the relative toxicity of A-series nerve agents, contrary to Mirzayanov's claim (Carlsen 2019). Using quantitative structure–activity relationship (QSAR) models, precisely the Toxicity Estimation Software Tool (T.E.S.T), the median lethal dose—LD_50_ value was calculated for oral administration to rats. The dose value was then converted to human. The acute toxicity (LD_50_ value) of A-series nerve agents was 5–75 times lower than the VX compound. Data were confirmed on the basis of the LD_50_ value for VX. For Ellison, it was 10 mg/person weighing 70 kg. When converted to mg/kg, the value is 0.14 mg/kg, which is consistent with Carlsen's calculated LD_50_ value for humans: 0.1 mg/kg (Carlsen 2019). Assuming the weight of a “standard” person was 70 kg, the LD_50_ values for the Novichoks chosen based on different data sources (Table 2) were estimated.

**Table 2:** Available data on toxicity of the Novichoks; based on (Karev 2009; Ellison 2016; Carlsen 2019)

Compound	LD_50_ (mg/kg bw)		
	(Karev 2009)	(Ellison 2016)	(Carlsen 2019)
VX	0.143	0.086–0.143	0.10
A-230	n.a	0.011–0.029	1.55
A-232	0.014–0.029	0.5	0.57
A-234	0.071	0.5	0.71
A-242	n.a	n.a	0.49
A-262	n.a	n.a	7.35

The hydrolysis half-lives of the A-series nerve agents were estimated at pH 6.5–7.4 using the QSAR Toolbox (Carlsen 2019). Moderate transformation due to hydrolysis, from 10 to 30 days, was shown by: VX, A-232, A-234 and A-262. The short half-lives of hydrolysis, less than one day, were shown by the following: A-230 and A-242 (Carlsen 2019). Suggests that, except for A-230 and A-242, the rest of the A-agents are relatively stable in the environment. The hydrolysis rate of Novichoks has also been measured experimentally at pH 7.2 at 25 °C (Harvey et al. 2020). The data showed that the hydrolysis of A-series agents was much slower by several orders of magnitude than G-series and V-series agents. Hydrolysis rate: GB = 6.68 μM/min, VX = 0.246 μM/min, A230 = 0.17 μM/min, A232 = 0.061 μM/min, A234 = 0.0032 μM/min (Harvey et al. 2020). The hydrolysis rate confirms the stability of Novichok compounds in the environment or in the organism. An additional group (Otsuka and Miyaguchi 2021) used density functional theory to perform theoretical calculations for the hydrolysis reactions of nerve agents, including Novichok compounds. According to data, A-series agents were as resistant to hydrolysis as VX and more resistant to hydrolysis than GB under basic conditions. The activation energy of hydrolysis under basic conditions is lower for compound A-234 compared to that under neutral conditions. Therefore, decontamination will be more effective under basic conditions (Otsuka and Miyaguchi 2021).

## Conclusions

Although Novichok is a relatively “hot topic”, there is still a lack of accurate, reliable data, and many unknowns require explanation (Bolt and Hengstler 2022). Establishing the structures and properties of Novichoks using theoretical tools (Jeong and Choi 2019) such as QSAR methods and adductomics (Golime et al. 2019; Sabbioni and Day 2022) is a promising field of study and necessary to understand the dangers these compounds can generate and develop adequate protection. Understanding the appropriate structures will enable theoretical and experimental research to discover the appropriate antidote. The synthesis of A-series compounds would enable the assessment of their toxicological and physicochemical properties. It would also create the possibility of developing modern methods for detecting these extremely toxic xenobiotics, such as characterization and study of the fragmentation pathways of Novichoks in aqueous solution by LC–MS/MS (Lee et al. 2021). On the basis of collected data concluded that Novichoks, also called A-series compounds, should be treated by analogy as other nervous factors, distinguished into the groups of compounds: G, V, and A. Novichoks should not be treated as independent groups of chemical warfare compounds. The new types of nerve agents constitute a constant and enormous danger. Although until now they have been used for assassination, their extreme toxicity poses a severe threat. Therefore, the danger caused by the Novichoks must be urgently assessed to be able to deal with future terrorist attacks or the use of chemical weapons on the battlefield. Improving and modifying international regulations and verification is necessary to prevent a catastrophic scenario using the A-series compounds. A crucial phase was the modification of the CWC regulations after the poisoning of Sergei Skripal and his daughter, which resulted in the introduction of some Novichok compounds into the treaty. Unfortunately, it did not cover all known A-series compounds, including their precursors used to form the Novichok binary forms previously included in the CWC list. Another significant modification is proposed in the introduction of Novichok agents with guanidine branches, which CWC does not cover. Results from Alexei Navalny's poisoning incident in 2020, most likely using the A-series compounds containing guanidine branches. Given the global danger posed by the usage of Novichoks, we believe that it will stimulate future progress to improve our protection against toxic agents and develop an optimal diagnosis and new treatments for casualties poisoned with these compounds. Furthermore, improve the lawful aspects of the CWC to reduce the likelihood of an attack. We hope that this review will inspire scientists to conduct future research to fill in gaps in missing data and highlight the dangerous potential of using Novichoks as a chemical weapon.

## Data Availability

For this review, we based on existing data from scientific articles.

## Abbreviations

ACh: Acetylcholine
AChE: Acetylcholinesterase
BChE: Butyrylcholinesterase
DCM: Dichloromethane
DF: Methylphosphonic difluoride
GB: Sarin
GD: Soman
GOSNIIOKhT: Institute for Organic Chemistry and Technology in Moscow
HuBuChE: Human butyrylcholinesterase
LD_50_: Median lethal dose
NNDA: *N*,*N*-Diethyl-2-iminopropan-1-amine
OP: Organophosphate
OPCW: Organization for the prohibition of chemical weapons
QSAR: Quantitative structure–activity relationship
Ser–His–Glu: Serin–histidine–glutamine
T.E.S.T: Toxicity Estimation Software Tool
TEA: Triethylamine
TEG: 1,1,3,3 Tetraethylguanidine
TMG: 1,1,3,3 Tetramethylguanidine
